# IgE-Mediated Anaphylaxis to Foods, Venom, and Drugs: Influence of Serum Angiotensin Converting Enzyme Levels and Genotype

**DOI:** 10.1155/2012/258145

**Published:** 2012-12-19

**Authors:** V. A. Varney, A. Warner, A. Ghosh, A. Nicholas, N. Sumar

**Affiliations:** ^1^Department of Medicine, St Helier Hospital, Wrythe Lane, Carshalton, Surrey SM5 1AA, UK; ^2^Department of Immunology, St Helier Hospital, Wrythe Lane, Carshalton, Surrey SM5 1AA, UK

## Abstract

Circulating angiotensin-II protects the circulation against sudden falls in blood pressure and is generated by the enzymatic action of angiotensin converting enzyme (ACE) on angiotensin-I. The ACE genes have 2 allelic forms, “I” and “D.” The “D” genotype has both highest angiotensin-II generation and serum ACE levels compared to “I”. 120 patients with IgE-anaphylaxis, 119 healthy controls, and 49 atopics had serum ACE levels, ACE genotype, and renin levels determined. Plasma renin levels were identical for all groups. Serum ACE levels and genotypes were similar for healthy controls (HC) and atopics, but lower in anaphylaxis (*P* = 0.012), with ACE genotypes also showing increased “I” genes (*P* = 0.009). This effect was more pronounced in subjects manifesting airway angioedema and cardiovascular collapse (AACVS) than mild cutaneous and respiratory (CRA) symptoms. AACVS was significantly associated with the presence of “I” genes. For “ID” genotype OR is 5.6, 95% CI 1.8 to 17.4, and for “II” genotype OR is 44, 95% CI 5 to 1891 within the anaphylaxis group = 0.001. The results show a difference in the genotype frequency between control and anaphylaxis, suggesting a role for the renin angiotensin system in anaphylaxis manifesting with airway angioedema and cardiovascular collapse.

## 1. Introduction

IgE mediated anaphylactic reaction can result in clinical symptoms ranging from mild cutaneous effects (Grade I) to cardiac arrest from profound hypotension and circulatory collapse (Grade 4) Terr classification [1]. To date we are unable to predict the severity of these reactions from the IgE levels alone or the allergen but the most severe reactions involve angioedema and hypotension. The effects of released histamine are central to this and produce falls in systolic blood pressure with angioedema from histamine effects on the microcirculation [2, 3]. Subjects tend to repeat their reactions on subsequent exposure unless the allergen dose is small [3, 4]. There are unanswered questions as to which host factors may be influencing the effects of the released histamine [5–7]. The renin angiotensin system (RAS) may be an important host factor. Angiotensin-II (AII) is the most potent vasoconstrictor that is rapidly generated in response to hypotension and protects against profound falls in systemic blood pressure such as those that occur in haemorrhage or shock [8–10]. 

**Figure 1:**
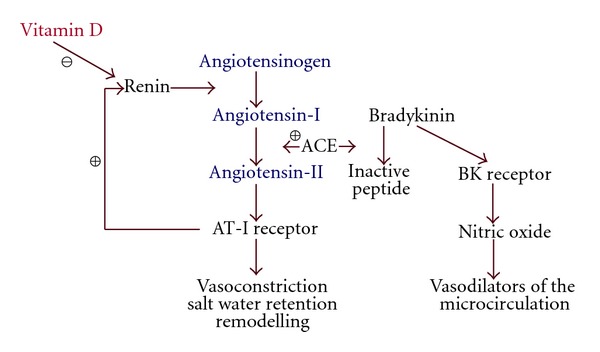
Schematic diagram of important interactions of renin angiotensin system.

The generation of AII from angiotensin-I (AI) is by the Angiotensin Converting Enzymes known as ACE, and is influenced by an individual ACE genotype [11–13]. Serum ACE also catabolises Bradykinin (BK) which influences the manifestation of angioedema in allergic disease [14–18] (Figure 1).

BK is a nonapeptide kinin formed from a plasma protein *α*_2_-globulin (kininogen) by the action of the enzymes kallikrein; it is a very powerful vasodilator that increases capillary permeability and angiooedema in allergic reactions via plasma extravasation from postcapillary venules [14, 15]. Typically this is mainly limited to the face, lips, tongue, and oropharynx including the upper respiratory tract. ACE is one of the most important enzymes for degrading BK in the circulation. BK's half-life is significantly shorter in subjects with a DD genotype due to the increased serum ACE activity of this genotype which reduces the effects of BK [16, 17]. 

The ACE gene exists in 2 allelic forms, with the presence of “I” or absence “D” of a 287 base pair intron of the gene [12, 19]. The deletion “D” genotype is associated with high AII generation and increased plasma BK catabolism [15, 18, 19]. In contrast, the insertion “I” is associated with lower ACE activity and impaired BK catabolism [14, 19, 20]. Individuals, who are homozygous for the D genotype (DD), have the highest serum ACE activity with higher plasma levels of AII [21]. BK degradation is also increased by high ACE activity [13, 22]. The “II” genotype is associated with reduced ACE activity which improves basal levels of BK with its effects on nitric oxide and vasodilatation of the microcirculation [15, 16].

 The heterozygous genotype ID follows the II genotype pattern for baseline ACE activity, plasma AII levels, renin activity, and blood pressure responses to exercise [18, 19, 23]. Renin activity reflects AI levels in the plasma and is the enzyme responsible for AI generation [23, 24]. 

In 1993 Hermann and Ring examined the RAS in patients with hymenoptera venom induced anaphylaxis and healthy controls [25]. Using Terr's classification [1] they demonstrated a significant reduction in the renin angiotensin hormones with increasing grade of anaphylactic severity (1–3). Their findings represented the first laboratory correlated parameter with the severity of clinical symptoms [26]. Recent studies of peanut and tree nut anaphylaxis have confirmed lower serum ACE concentrations, <37 mmol/L were 9.7X more likely to develop pharyngeal oedema [27]. ACE-inhibitors drugs have been associated with prolonged anaphylaxis [22, 28]. 

Vitamin D may have an influence in allergic diseases as suggested by Epi-Pen prescriptions being fourfold higher in the northern states of the USA [29]. Vitamin D does lower plasma renin levels by up to 50% [29, 30].

 In our study, we have examined plasma renin, 25 hydroxy vitamin D, serum ACE levels, and ACE genotype in 119 controls, 49 atopics, and 120 subjects with prior IgE mediated anaphylaxis. 

## 2. Methods

The study was performed at St Helier Hospital, Surrey with permission granted by our local regional Ethics Committee, and each patient gave written informed consent. The patients were enrolled for the study between 2006 and 2009. Sample sizes and power calculations were determined and approved by statistical advice as required by the Ethics committee. This gave a sample size minimum of 50 subjects for each group, which was comparable with other studies linking genotype to disease states. For the healthy controls and anaphylaxis groups in this study this number was exceeded. 

### 2.1. Subjects

 288 subjects were selected. 119 were healthy nonatopic controls who were medication-free. 49 were atopic with airborne allergens ± foods who developed only minor intestinal or oral symptoms but no anaphylaxis. 120 subjects were atopic with IgE mediated anaphylaxis on one or more occasions in the past to food, venom, or drugs. No subjects were taking ACE inhibitors or angiotensin receptor antagonists.  Table 1 shows the subject demographics and allergen sensitivity.

### 2.2. Clinical Assessment

Subjects were selected from the allergy clinic at St Helier Hospital. All subjects including healthy controls (HC), were skin prick tested to airborne allergens and food to confirm and establish their exact of atopic status. Detailed records of allergic history, past medical history, drug medication, and family history were recorded in all subjects.

### 2.3. Assessment of Anaphylaxis

All reactions were recorded in detail with IgE mediated causes proved by skin tests, ± specific IgE, in addition to history. The route of allergen exposure, time to onset of symptoms, duration, and treatment were recorded. Symptoms were scored in an identical fashion using a pro forma (Table 5), with each symptom enquired about from the check list. This permitted grading of the anaphylaxis from Grade I (mild systemic) to Grade 4 (cardiac arrest). The check list used in the study is presented in Table 5. 

Further subdivisions reflecting likely ACE symptoms were made into the following:

1. those with airway angioedema and cardiovascular collapse (AACVS),
2. those with cutaneous respiratory symptoms only (CRA).

Subjects in the AACVS group had suffered symptomatic hypotension or syncope in all previous anaphylactic reactions, with some subjects describing prolonged unconsciousness and 5 subjects having a cardiac arrest. In addition most of these subjects (>95%) developed airway swelling affecting mouth, tongue, or throat. Since these symptoms would relate more directly to likely ACE activity, they were analysed as a separate group and related to their genotype.

Subjects in the CRA group described all previous reactions to consist of chest symptoms (bronchospasm, cough, and wheeze) with pruritus, urticarial, and conjunctivitis also present to varying degrees. In some abdominal cramps ± vomiting and diarrhoea had occurred. These manifestations were considered to related more to histamine release than ACE activity. No subject in this group described angiooedema or hypotensive symptoms in any of their previous reactions.

### 2.4. Total Serum IgE and Allergen Specific IgE

These were measured in all cases of hymenoptera venom allergy and drug induced anaphylaxis, where the information was combined with skin prick testing and intradermal testing as appropriate. The food induced anaphylactic subjects had IgE and specific IgE tests only if strong confirmation by skin prick test was judged unsatisfactory. Since IgE levels were only measured where indicated, no subsequent analysis involving IgE for the cohorts could be made.

Most measurements were performed in house by our Immunology Laboratory Department at St Helier by Phadia 250 Unicap analysis. 

### 2.5. Skin Prick Tests

A range of products were used, Alk-Abello for airborne allergens and hymenoptera venom, and Hollister Stier for food allergens with “fresh food” tests in some subjects. Skin prick tests were read at fifteen minutes with appropriate positive and negative controls, and without antihistamine use for five days.

### 2.6. Serum ACE Blood Samples

Blood samples for serum ACE were collected as a morning fasting sample with the subject supine for at least 20 minutes. The samples were collected in tubes at 4°C and spun in a refrigerated centrifuge at 4°C for 10 minutes at 4500 g. The serum was removed into labelled tubes at 4°C and rapidly stored at −20°C until analysis at the end of the study.

### 2.7. Serum ACE Measurements

Measurement was performed by our Biochemistry department, St Helier Hospital, using the Bulmann ACE kinetic test kits. This measures the cleavage of a synthetic substrate (FAPGG) by the ACE enzyme, which is measured by absorbance at 340 nm. Quality controls and standard curves were plotted. The normal ranges for serum ACE activity were 20–70 U/L with variation in ranges known to reflect ACE genotypes variations.

### 2.8. Hardy-Weinberg Equilibrium

The ACE gene polymorphisms follow Mendelian characteristic as predicted by the Hardy-Weinberg equilibrium and described by Butler et al. [31] and Rigat et al. [32].

### 2.9. Blood Samples for ACE Genotype

 EDTA samples were collected and centrifuged for ten minutes at 4500 g. Plasma was removed without disturbing the buffy coats. The plasma samples were then frozen at –20°C until the study was completed. The buffy coats were then used for DNA extraction.

### 2.10. Isolation of Genomic DNA

Genomic DNA was subsequently prepared from the buffy coats (300 *μ*L) using the Promega Maxwell 16 semiautomated extraction system (Promega). The extracted DNA samples were quantified (Eppendorf Biophotometer) and stored at −20°C until ready for PCR.

### 2.11. ACE Gene Polymerase Chain Reaction and Identification of Genotype

 The PCR reaction was set up in a total volume of 25 *μ*L with 20 *μ*L of Reddy Mix PCR Master Mix (ABgene Ltd, UK, Thermo Scientific) containing 1.5 mM MgCl_2_, 20 mM ammonium sulphate in 75 mM Tris-Hcl buffer and 50 pmol of the forward and reverse oligonucleotide primer (MWG Biotech, Germany), and 5 *μ*L of 50–100 ng of genomic DNA template.

The sense and antisense primers had the following sequences [33]:

5′ CTGGAGACCACTCCCATCCTTTCT 3′

5′ GATGTGGCCATCACATTCGTCAGA 3′

PCR cycling conditions were heating at 94°C for 2 min followed by amplification for 35 cycles; each cycle consisted of denaturation at 94°C for 1 min, annealing at 55°C for 30 sec, and extension at 72°C for 90 sec.

The PCR products (10 *μ*L) were separated on 2% agarose gel with ethidium bromide using TBE buffer solution. A 100 bp DNA ladder size marker (8 *μ*L) was used. The amplified PCR products were visualised as bands under UV light. I and D alleles were identified at 490 bp and 190 bp, respectively. 

Samples identified as DD were subjected to a second round of PCR amplification using insertion specific sequence as described by Tomita et al. (1997) [34].

This PCR identified a 335 bp in the presence of the I allele but not in DD homozygotes. 

### 2.12. Measurement of Plasma Renin Levels

Measurement was performed by ELISA kit (E90889Hu) supplied by USCNK life science, China. Normal plasma levels of renin range were 1130–2920 pg/mL resting supine (J Physio 2011; 589 : 1272-81).

### 2.13. Measurement of Vitamin D Levels

25-Hydroxycholecalciferol was measured by our Biochemistry department [35] using the IDS iSYS analyser. Replete levels are 75–200 nmol/L, severe deficiency <25 nmol/L.

### 2.14. Statistical Analysis

Statistical analysis was carried out using Sigma Stats 3.1 version statistical package. The level of significance was adjusted in Bonferroni fashion for multiple comparisons predetermined by the number of comparisons using the Bonferroni correction to avoid false positive findings, which for 3 main groups comparison would give a statistical significance at (0.05/3 = 0.016)*P* = 0.016. 

### 2.15. Mean Serum ACE levels

With SEM, SD, and 95% CI were calculated for the 3 groups. Between groups comparisons were made by ANOVA testing and unpaired *t*-tests. 

### 2.16. ACE Genotype Analysis

Genotype profiles of all the groups and subgroups with different grades of anaphylaxis were compared by Pearson's chi-square test for categorical data. chi-square test was used for assessment of the Hardy-Weinberg equilibrium for the distribution of genotypes.

The analysis was performed for all 288 patients as a group. 

The genotype frequency for atopics and anaphylactic groups were firstly compared with healthy controls using a chi-square test (2df). Odds ratios were calculated with 95% confidence intervals comparing the presence of ID and II with DD. Genotypes in anaphylactic subjects with AACVS were compared with those with CRA, and also for traditional anaphylaxis Grades 1–4.

### 2.17. Plasma Renin Levels

Mean plasma renin levels in pg/mL were calculated for the groups and compared with the ACE genotypes and AACVS/CRA subdivisions by ANOVA and unpaired *t*-test. Normal basal resting range was 1130–2920 pg/mL.

### 2.18. 25-OH-Vitamin D Analysis

Mean levels (nmol/L) were calculated for the groups and comparisons made by ANOVA and unpaired *t*-test with the plasma renin levels taken at the same time. Normal range was >75 nmol/L.

## 3. Results

Demographic Information (Table 1) shows the demographics of the 3 main groups including allergen sensitivity and anaphylaxis grading 1–4.

The mean ages are similar and do not influence the genotype or serum ACE levels. Allergic disease also has a female predominance, with a female/male ratio of 1.35 reported in the UK [2].

Table 2 serum ACE levels shows that all groups fell within the normal range (20–70 U/L). HC and atopics had a similar mean (48 U/L), while the anaphylaxis group was significantly lower (33.2 U/L). Within the CRA and AACVS subgroups, both remained statistically lower than HC and atopics. The serum ACE differences between the groups were confirmed by ANOVA testing and individual *t-*tests with significance at the *P* = 0.016 level. This confirmed serum ACE levels were significantly lower in the anaphylaxis group and AACVS subdivision compared with HC and atopics. From work related to serum ACE levels and sarcoid, it has been demonstrated that DD genotypes occupy the upper end of the normal range while II genotypes give serum values at the lower end [34]. Our results probably reflect this observation.

Table 3, ACE genotypes, shows the genotype frequency distribution including chi-squared analysis and *P* values. Among the healthy controls, the gene frequency was consistent with European population data (EU data of general population DD 43%, ID 39%, II 18%). There were no statistical differences between HC and atopics, although atopics had a higher percentage of ID gene frequency suggesting that they may be half way between normal and anaphylaxis. As discussed later it could be due to the smaller atopic group size. Anaphylaxis patients were different with a predominance of ID and II genes, and a reduction in DD. This difference was most marked in the AACVS group. Compared with atopics, CRA showed increased DD genotype frequency and AACVS showed increased II gene frequencies- but the *P* values did not reach the Bonferroni adjusted *P* value of 0.016. We did not find any definite associations between genotype and specific allergen (e.g., nuts, shellfish, venom, etc.); the correlation was with genotype alone.

### 3.1. ACE Genotype and Relationship to Grade 1–4 Anaphylaxis, Table 3

This grading system does not clearly distinguish between CRA only symptoms and AACVS, because these symptoms are mixed together in each grade (Table 5). The chi-squared analysis did not show a significant difference between the 4 grades.  Table 4 analysis of gene frequencies-, shows that anaphylaxis patients were more likely to be an ID genotype (odds ratio 3.3) or II genotype (odds ratio 3.1) than DD, *P* = 0.001. Atopic controls showed a similar tendency towards a lower prevalence of DD genotype but this did not reach significance (*P* = 0.082). While II genotype was more common among AACVS than the healthy controls, none of the CRA subjects had II genotype although they showed a frequency of DD genotypes similar to the healthy controls.

There was a strong relationship between anaphylaxis and genotype compared with healthy controls, which is due to AACVS group, with DD being more common in healthy controls. CRA subjects have fewer II than healthy controls, atopic subjects, or AACVS.

Atopic subjects also have less DD than healthy controls, but this is not significant. Anaphylaxis subjects have less DD than atopics but this is not significant. However the atopic group is relatively small and this data suggests the atopics fall between anaphylaxis and healthy controls but there is insufficient data to show significant results.

### 3.2. Analysis of Renin Levels and Vitamin D Levels

Mean plasma renin levels in pg/mL were identical between the groups, which was confirmed by ANOVA as well as individual *t-*tests. HC = 3647, atopics = 3553, CRA = 3510, and AACVS = 3570, suggesting that renin did not influence our findings. Mean serum 25-OH vitamin D levels were in the “insufficient range of <75 nmol/L” for all groups which may represent our UK climate and latitude.

HC had higher mean vitamin D levels 54.3 nmol/L ± 26 SD than atopics 44.8 nmol/L ± 23 SD (*P* = 0.032, 95% CI −18.1, −0.89), or anaphylaxis; mean was 46 nmol/L ± 19 SD (*P* = 0.020, 95% CI −15.1, −1.32), but these did not reach the Bonferroni adjusted *P* value of 0.16. Vitamin D insufficiency may obscure any expected relationship between it and renin levels. A scatter plot showed no significant relationship between the values. 

## 4. Discussion

In the UK, 2% of the adult population carry adrenaline due to prior anaphylactic reactions [2]. The genes for Atopy, bronchial hyperresponsiveness, and asthma are being recognised and form a polygenetic pattern of inheritance but there are no links between individuals with anaphylaxis and any specific “allergy genes” [36]. Current research is examining food allergy and conformational and sequential antibody-antigen binding sites [37]. This may ultimately confirm a difference in IgE binding which may affect levels of histamine release and the manifestation of these reactions.

Our study demonstrates a link between ACE genotype and anaphylaxis to food, venom, and drugs, particularly for reactions that include cardiovascular collapse and severe angioedema. Here the genotype is ID and II relative to HC. These two genotypes share similar ACE activity and AI and AII plasma levels and physiological responses [14, 15, 18] and are therefore likely to behave in a similar manner clinically in response to hypotension and BK metabolism [12, 15, 18, 23]. The AACVS pattern of symptoms would be very consistent with low levels of ACE activity, producing both inadequate or delayed A2 generation to prevent hypotension and impaired BK metabolism that could facilitate angioedema [11]. 

Few patients with the DD genotype were found in the anaphylactic group, and most of those manifested cutaneous and respiratory symptoms only, which are least likely to be associated with reduced ACE activity and more likely to be related to histamine effects [11, 19]. The CRA group only represented 23% of the total group and so may be subject to bias.

The atopic group is smaller than the other groups and so may lack sufficient power to examine fully its relationship with healthy controls. Atopics do show an increased ID genotype population although this was not significant (*P* = 0.14), but may suggest that they lie somewhere between HC and anaphylaxis and warrants further investigation.

Niedoszytko et al. [38] examined angiotensinogen (MM/MT) and ACE gene (I/D) polymorphisms in 107 patients with Grades III and IV anaphylaxis to insect venom and 113 controls. They found no difference in ACE genotypes between subjects and controls. Neither could they relate it to the conventional grades of anaphylaxis. In addition they identified polymorphisms in the angiotensinogen gene M235T resulting in low levels of angiotensinogen in 29.9% of subjects compared with 17% of controls (*P* = 0.02). In grade IV anaphylaxis this frequency increased to 39% (*P* = 0.001, odds ratio 2.5). Since this is the substrate for renin to generate AI this could be an important additional genetic factor in anaphylaxis.

Our serum ACE levels appear to reflect the genotype findings, with HC and atopics being similar, but anaphylaxis showing significantly lower levels especially for the AACVS subdivision. These findings fit well with those of Summers et al. [27] who examined serum ACE levels in patients with tree/nut allergy. He found that mean ACE levels above 47 mmol/L were not associated with pharyngeal oedema, while serum ACE levels <37 mmol/L were a risk factor for pharyngeal oedema but not asthma. He suggested that since ACE levels are stable in adult life and independent of age or sex, they reflect genetic inheritance. 

In women of reproductive age it is now understood that oestrogen protects against cardiovascular disease due to the inhibitory effects of oestrogen upon mRNA synthesis of ACE [39]. This protective effect contributes to the hypotension of pregnancy as oestrogen levels climb and is lost at menopause when serum ACE levels rise unless inhibited by the use of hormone replacement therapy [40–42]. This could suggest that premenopausal women effectively “inactivate a “D” gene,” through their oestrogen effects on ACE synthesis, rendering themselves more ACE deficient than men for the same genotype [39]. This could offer one explanation as to why adult premenopausal women show an increased anaphylaxis rate compared to men; an observation which is lost after menopause. 

Anaphylaxis research in rodents suggests that platelet activating factor (PAF) is involved in cardiovascular collapse in murine models of anaphylaxis which can be prevented by PAF antagonists [43, 44]. In PAF knockout mice, no hypotension or death in anaphylaxis occurs. PAF levels have been measured in man, and are reduced immediately following anaphylaxis [45, 46], but other data indicates that these levels are normal if measured away from the event. PAF maybe acting through endothelial nitric oxide (eNO), when mediating its hypotensive effect in anaphylaxis [4, 43, 45]. eNO is now of interest in murine models of anaphylaxis, where chronic blockade of eNO increases expression of mRNA for renin, ACE, and AI receptors in the aorta, with the risk of hypertension [47, 48]. In man eNO is involved in regulation of the microcirculation but has links with RAS, BK, and renal function [6, 10, 48]. Activation of RAS enhances eNO production, giving vasodilator effects on the microcirculation to protect against the vasoconstrictor effects of AII [31]. Oestrogens have been shown to increase eNO levels and protect the circulation leading to the better renal function and resistance to renal injury observed in women [7, 47]. Basal AII levels exert a tonic effect on the circulation which reduces eNO [8]. This may suggest that individuals with DD genotypes, and therefore higher basal levels of AII, would be more protected from the risk of cardiovascular collapse via eNOS and PAF in anaphylaxis [15, 31, 39, 47, 49]. This may not be true for ID and II genotypes especially in premenopausal women, where the presence of oestrogen reduces basal AII levels giving higher levels of BK and eNOS. Vitamin D has effects on renin levels and several publication suggest a link to allergy, anaphylaxis, and Epi-Pen prescription [29, 50]. Renin synthesis is lowered by vitamin D at the mRNA level and is suggested to be cardioprotective in this way [30, 51–54]. Lowering levels of renin would theoretically reduce its enzymatic activity and AI generation which may reduce eNOS productions. This observation is probably minimised in our study, since nearly all subjects had vitamin D insufficiency, although the atopics and anaphylaxis groups had the lowest levels. Further work in this area would be of interest.

## 5. Conclusion

Our results suggest that anaphylactic reactions to foods, venom, and drugs which involve acute angioedema and cardiovascular collapse may have links with II and ID ACE genotypes and lower serum ACE levels, which is less clearly related to the conventional anaphylaxis Grades I–IV. Serum ACE levels may become useful in anaphylaxis since it reflects genotype. Oestrogen and its effects on serum ACE levels may explain the difference in anaphylaxis rates between men and women with further research of interest including the links between the renin angiotensin system to eNO and PAF. 

## Figures and Tables

**Table 1:** Demographics of study groups.

Subject groups	Mean age (range)	% Female	Allergen sensitivity number of cases	Grades 1–4 (no. of cases)
Healthy controls *N* = 119	53 (25–71)	73		

Atopic controls *N* = 49	47 (18–77)	79	Fish 2 Dairy 3 Fruit/nut 12 Airborne 32	

Anaphylaxis *N* = 120	45 (16–71)	65	Venom 44 Nut/fruit 35 Fish 14 Cereals 5 Drugs 6 Exercise 4 Latex/others 12	Grades (G) G1 = 10 G2 = 54 G3 = 51 G4 = 5

**Table 2:** Serum ACE levels for the study groups.

Subject groups	Mean serum ACE levels U/L ± SD	*P* value V's HC (95% CI)	*P* value V's atopic (95% Cl)	*P* value V's anaphylaxis group *n* = 120 (95% CI)
Healthy controls *N* = 119	48.9 ± 25		0.53	0.012 (3.4, 27.9)
Atopic controls *N* = 49	47.9 ± 25	0.86		0.018 (−16.1, −1.5)
Anaphylaxis *N* = 118	33.2 ± 20	0.012 (3.4, 27.9)	0.018 (−16.1, −1.5)	
CRA* *N* = 27	35.1 ± 25	0.031 (1.14, 24.3)	0.025 (1.6, 25.9)	0.23
AACVS^+^ *N* = 93	31.2 ± 20	0.015 (1.72, 15.5)	0.011 (2.2, 17.1)	0.81

*Cutaneous and respiratory allergy.

^+^Acute angioedema and cardiovascular collapse.

**Table 3:** ACE genotype frequency for the groups and anaphylaxis grades.

ACE genotype	DD	ID	II	Healthy controls	Atopics	Anaphylaxis	Cutaneous and RS
Healthy controls (HC) *N* = 119	53 (45%)	44 (37%)	22 (18%)		*P* = 0.14	*P* = 0.009	*P* = 0.053
Atopic controls *N* = 49	15 (31%)	26 (53%)	8 (16%)	*P* = 0.14		*P* = 0.42	*P* = 0.025
Anaphylaxis *N* = 120	30 (25%)	58 (50%)	32 (25%)	*P* = 0.009	*P* = 0.42		
Cutaneous & respiratory anaphylaxis *N* = 27	15 (56%)	12 (44%)	0 (0%)	*P* = 0.053	*P* = 0.025		
Airway angioedema ± CVS collapse *N* = 93	15 (16%)	46 (49%)	32 (35%)	*P* < 0.001	*P* = 0.024		*P* < 0.001

Traditional grades of anaphylaxis	DD	ID	II	Grade 1 *P* value	Grade 2 *P* value	Grade 3 *P* value	

Grade 1	4 (40%)	5 (50%)	1 (10%)		*P* = 0.12	*P* = 0.78	
Grade 2	9 (17%)	25 (46%)	20 (37%)	*P* = 0.125		*P* = 0.048	
Grade 3	16 (31%)	26 (51%)	9 (18%)	*P* = 0.783	*P* = 0.048		
Grade 4	1 (20%)	2 (40%)	2 (40%)	*P* = 0.373	*P* = 0.96	*P* = 0.48	

**Table 4:** Odds ratio analysis of genotype comparison with DD genotype.

Comparisons with DD	Odds ratio (95% CI)	Odds ratio (95%CI)	Chi square *P*-value
	ID	II	
Relative to healthy controls (*n* = 119)			
Atopies (*n* = 49)	2.3 (1.0–5.4)	1.4 (0.4–4.1)	0.082
Anaphylaxis (*n* = 120)	3.3 (1.7–6.3)	3.1 (1.4–6.9)	<0.001
CRA (*n* = 27)	1.1 (0.4–2.9)	0 (0–0.7)	0.051
AACVS (*n* = 93)	6.3 (2.7–15.3)	7.5 (2.9–19.8)	<0.001

Relative to atopic subjects (*n* = 49)			
Anaphylaxis (*n* = 120)	1.4 (0.6–3.3)	2.3 (0.7–7.3)	0.24
CRA (*n* = 27)	0.5 (0.2–1.5)	0 (0–0.5)	0.028
AACVS (*n* = 93)	2.7 (1.0–7.7)	5.4 (1.5–19.5)	0.009

Relative to CRA (*n* = 27)			
AACVS (*n* = 93)	5.6 (1.8–17.4)	44 (5–1891)*	<0.001

*calculated by adding 1 to each cell, biased towards 1.

**Table 5:** Check list used for conventional anaphylaxic symptoms Grades 1–4.

Anaphylaxis grades	1	2	3	4
Hypotension	Faint/dizzy	Unconscious	Prolonged unconsciousness	Cardiac Arrest
Bronchospasm	Wheezy	Severe	Poor response to treatment	
Angioedema	Lips/face	Generalised	Severe	
Airway oedema	Hoarse	Difficulty in swallowing	difficulty in breathing	
Urticaria	Mild	General	severe	
Pruritus	Mild	Severe		
Conjunctivitis	Mild	Severe	Ulcerated	
Vomiting	Once	Severe	Prolonged	
Diarrhoea	Once	Severe	Prolonged	
Abdominal	pains	Severe cramps	Bleeding	
CNS	Describe all			

## Abbreviations

ACE: Angiotensin converting enzyme
HC: Healthy controls
AACVS: Airway angioedema and cardiovascular collapse
CRA: Cutaneous and mild respiratory anaphylaxis
BK: Bradykinin
AI: Angiotensin-I
AII: Angiotensin-II
eNO: Endothelial nitric oxide
mRNA: Messenger ribonucleic acid
RAS: Renin angiotensin system
PAF: Platelet activating factor
L NAME: N (G)-nitro-L-arginine methyl ester.
